# The formation mechanism of tear strips on stretched Ti-22Al-25Nb alloy sheets

**DOI:** 10.1038/s41598-017-01889-9

**Published:** 2017-05-10

**Authors:** Yingying Zong, Bin Shao, Wenchen Xu, Bin Guo, Debin Shan

**Affiliations:** State Key Laboratory of Advanced Welding and Joining, Harbin Institute of Technology, Harbin, 150001 China

## Abstract

This paper reports the presence of tear strips on the surface of a Ti-22Al-25Nb alloy sheet stretched at 960 °C. The test piece reveals a “bamboo”-shaped pattern on its surface, which severely affects the quality of the alloy. Microstructure analysis indicates that the formation mechanism of the tear strip is related to both the rich α_2_ phase layer and the interfacial B2 phase dynamic recrystallization layer between the α_2_ phase layer and the substrate metal.

## Introduction

Ti-22Al-25Nb alloy is a second-generation Ti_2_AlNb-based alloy^1^ that is composed of a body-centred cubic B2 phase, an orthogonal structure O phase, and a hexagonal closely packed α_2_ phase. The B2 phase is the substrate of the alloy and is vulnerable to sliding deformation, demonstrating good plastic deformation^2, 3^. The O and α_2_ phases strengthen the alloy. The Ti-22Al-25Nb alloy is characterized by high strength, light weight, and high temperature resistance and is a new generation of aerospace structure material^4–6^. However, its extensive application has proven challenging due to difficulties in its hot forming. Therefore, a large number of studies have been conducted to study the tensile, compressive and processing properties of the Ti-22Al-25Nb alloy^7–10^. The research results reveal good plastic deformation capability of this alloy at temperatures greater than 950 °C^11^. In general, the elongation rate exceeds 80%, and the deformation resistance is smaller than 200 MPa^12^. In addition, dynamic recrystallization is observed during the deformation of this alloy^13, 14^. The alloy is suitable for hot forming under such conditions^10, 15–17^. Most forming devices do not have a good protection environment in real production processes, which leads to oxidation of this alloy at elevated temperature, generating oxides such as TiO_2_, AlNbO_4_, and Al_2_O_3_. Oxidation results in a sequence of surface defects, such as surface roughness and cracking, affecting the surface quality of the alloy^18, 19^. The Ti-22Al-25Nb alloy is primarily used in aviation spacecraft in the form of sheet metal, such as mounting frames for insulation and the exterior surface^20, 21^. The surface quality of the metal sheet is an important quality indicator of the workpiece. Thus, it is important to determine the surface defects during the forming process and to develop ways to avoid surface defects and improve the surface quality of the alloy components.

## Materials and Experimental Procedures

**Figure 1:**
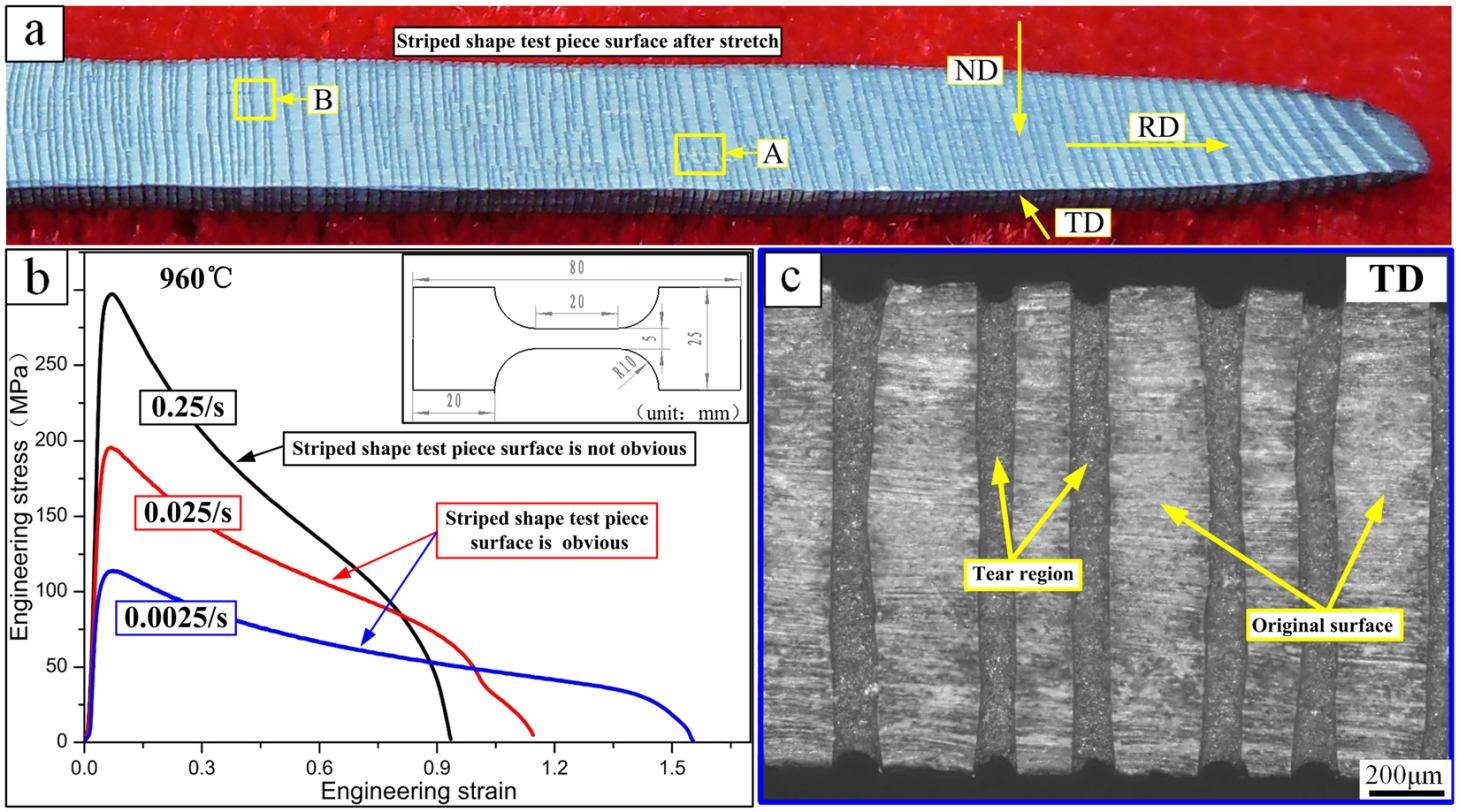
Stretched surface tear region; (**a**) macroscopic morphology after test piece fracture; (**b**) stress-strain curve; (**c**) TD surface “bamboo”-shaped tear region of the specimen in Fig. 1(a).

The material used in this report was a 1 mm thick Ti-22Al-25Nb alloy sheet. The tensile test was performed with an Instron-5500R electronic universal testing instrument. The gauge length and width of the tensile test piece were 20 mm and 5 mm, respectively, as shown in Fig. 1(b). The stretching temperature was 960 °C, and the strain rates were 0.0025/s, 0.025/s, and 0.25/s. Before stretching, heat preservation was performed for 15 min, and water cooling after the fracture process maintained the tensile structure. The specimen was then polished by electrolysis for microstructure observation. The metal phase structure was observed using an Olympus GX71 optical microscope. Back-scattered electron microscopy (BSEM) and electron back-scattered diffraction (EBSD) were also employed, using a Supra 55 Sapphire SEM. The Supra 55 Sapphire SEM, equipped with an INCAEnergy detector and an INCACrystal/HKL detector, was used for composition analysis and EBSD signal collection. The software Project Manager was used to analyse the EBSD data. The hardness testing device was a HVS-1000Z digital micro hardness tester.

## Results and Discussion

Obvious stripes are generated at the alloy surface during the tensile experiment as the temperature exceeds 960 °C. Thus, the specimen stretched at 960 °C is examined to reveal the cause of the stripes. As shown in Fig. 1(a), the ND and TD surfaces of the test piece show numerous stripes. Figure 1(b) shows the stress-strain curve of the alloy deformed at 960 °C and 0.0025/s~0.25/s. The experiment results indicate that obvious stripes are generated at the alloy surface when the strain rate is less than 0.025/s. The elongation rates under these conditions exceed 110%. Optical microscope observations reveal that the stripes possess a stripped type of surface tear (Fig. 1(c)). The tear strip depth is approximately 30 μm, with a width of approximately 100 μm. Following deformation, the test piece demonstrates a “bamboo” shaped structure, which severely affects the surface quality of the test sample. The analysis indicates that the substantial deformation of the alloy is one of the main causes of the surface tear.

**Figure 2:**
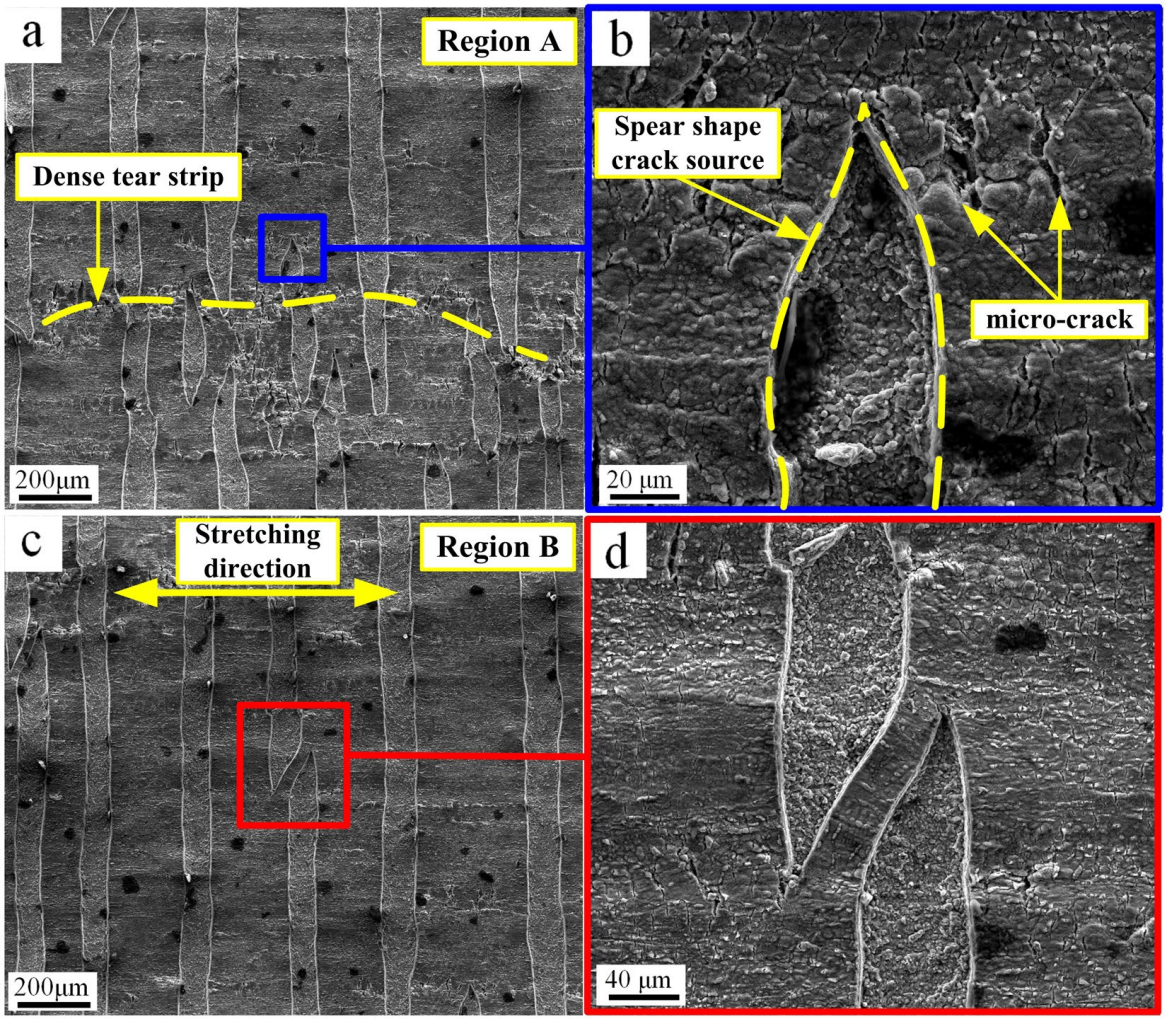
ND surface tear strip; (**a**) SEM morphology of region A in Fig. 1(a); (**b**) tear source morphology; (**c**) SEM morphology of region B in Fig. 1(a); (**d**) tear strip intersection.

Figure 2 shows the ND surface tear region, and Fig. 2(a) is a magnified version of region A in Fig. 1(a). The tear strip shows a straight vertical shape perpendicular to the stretching direction. Dense tear sources are produced in the stretching direction because of local deformation unevenness, as indicated by the yellow line in Fig. 2(a). As shown in Fig. 2(b), the tear source end possesses a “spear” shape, and the oxidized skin has a large number of micro-cracks. In addition to the straight strip tears, Fig. 2(c) shows that crossing tear regions are also present, which are magnified in Fig. 2(d). This result suggests that the tear expands towards the front spear end after a tear source is formed. Moreover, the shape in Fig. 2(d) forms when two tear sources expand from two ends of the test piece and intersect at the centre. Therefore, the formation process of the surface tear strip involves the following three steps. First, surface tears form micro-cracks that gradually expand to form a spear tear source. Second, stress is concentrated at the front end of the spear shape and continuously expands frontward. Third, the tear strip terminates once it expands to the test piece edge or when two tear strips meet, forming a stripe-shaped tear strip.

**Figure 3:**
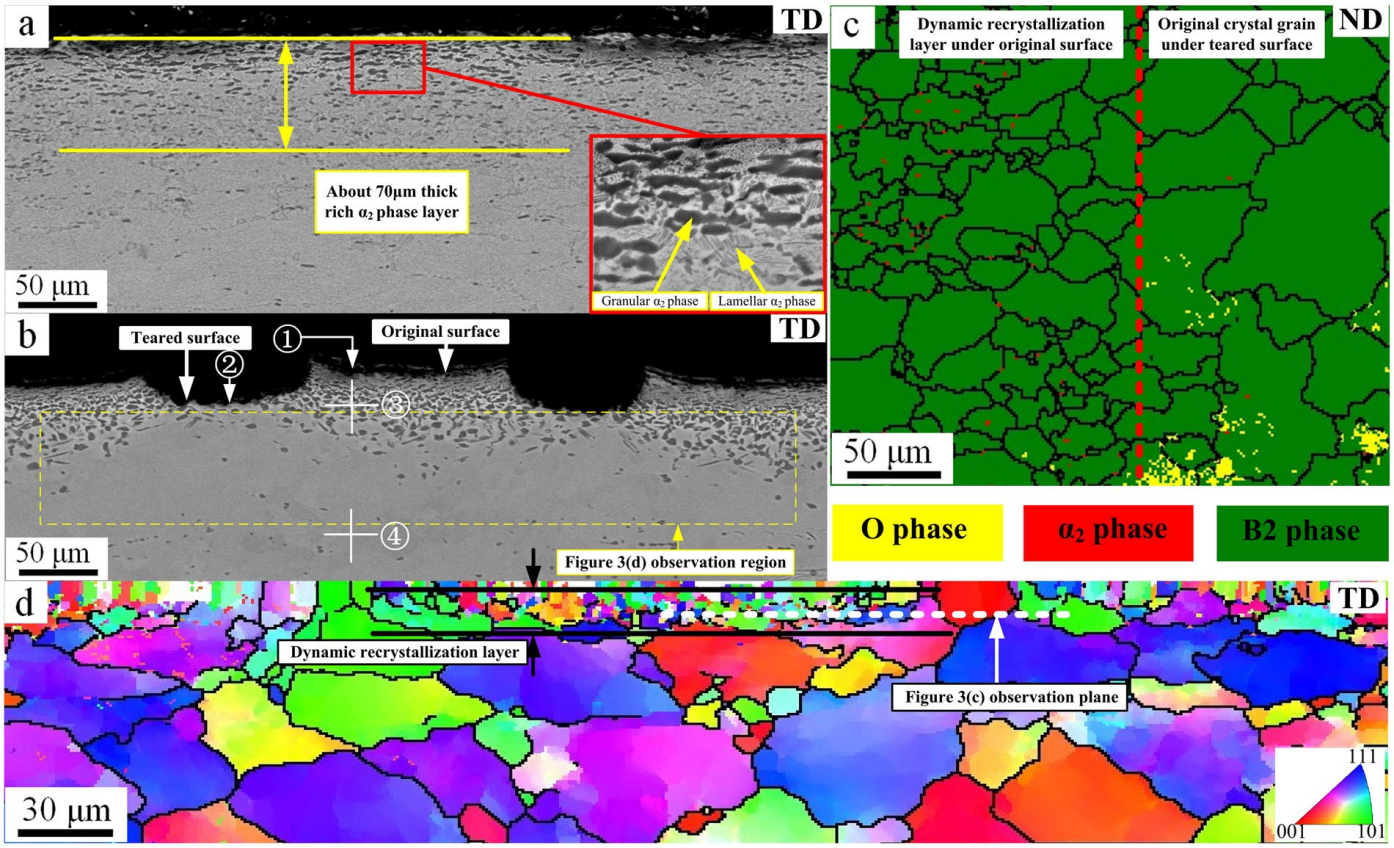
Stretching microstructure; (**a**) TD surface via BSEM prior to stretching; (**b**) TD surface via BSEM after stretching; (**c**) ND surface dynamic recrystallization layer; (**d**) EBSD orientation distribution of the yellow region in (**b**).

The α_2_ phase distribution in the test piece is not uniform before the alloy is stretched at 960 °C. The α_2_ phase is rare in the substrate region but is abundant near the surface, forming a rich α_2_ phase layer with a thickness of approximately 70 μm. Granular α_2_ phase and lamellar α_2_ phase exist in the rich α_2_ phase layer, as shown in Fig. 3(a). The lamellar α_2_ phase spheroidizes and grows during the tensile process^22^, and the rich α_2_ phase layer tears during the tensile process, as shown in Fig. 3(b). Table 1 shows the composition and hardness of the alloy at locations ①, ②, ③ and ④ in Fig. 3(b). The oxygen content gradually decreases from the outer skin at ① to the inside of the alloy at ④. The oxidation products are primarily TiO_2_ and Al_2_O_3_ at ①^23, 24^ and TiO_2_ at ②. Additionally, the rich α_2_ phase layer is rich in Al and O but lacks Nb, which provides favourable conditions for growth of a rich α_2_ phase. The hardness of the rich α_2_ phase layer (450 HV) is significantly higher than the hardness of the B2 phase substrate (335 HV). Overall, this scenario causes uneven deformation, resulting in surface tears during large plastic deformation.

**Table 1:** Content and hardness of the regions indicated in Fig. 3.

Location		Content (At.%)			
		Ti	Al	Nb	O
①		22.10	12.69	07.58	57.63
②		25.33	10.32	12.71	51.64
③		53.47	21.03	15.32	10.18
④		51.15	19.21	24.83	4.81
Hardness	Region ③	450	447	452	Average 450
	Region ④	335	330	339	Average 335

**Figure 4:**
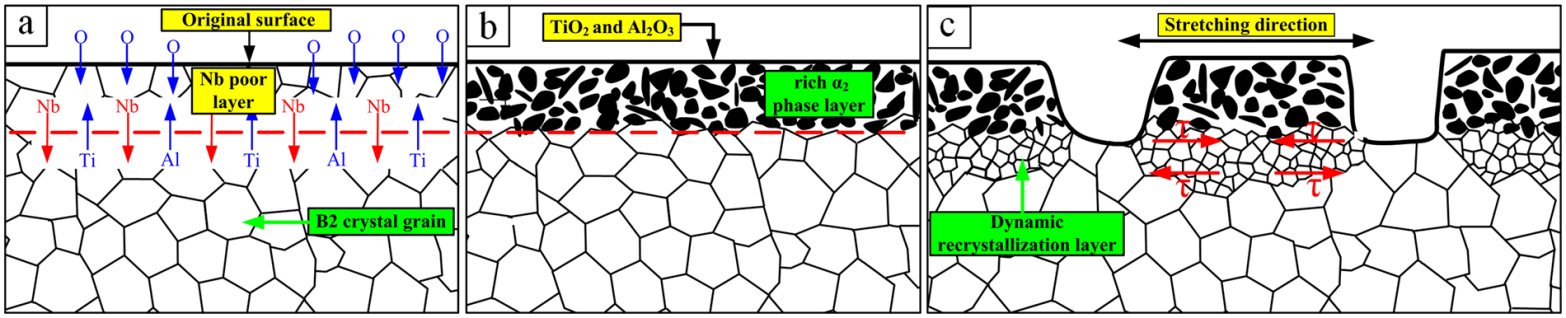
Formation mechanism of tear strips; (**a**,**b**) Rich α_2_ phase layer formation process; (**c**) Tear strip formation process.

If only the rich α_2_ phase layer and the core B2 phase substrate existed, the tear cracks on the alloy surface after stretching would have a dispersed distribution and would not form a stripe-shaped tear strip. Another main cause of the formation of the stripe-shaped tear strip is shown in Fig. 3(d). The high hardness of the rich α_2_ phase layer restricts the deformation of the neighbouring B2 phase layer, producing shearing stress on this layer. The deformation is severe and occurs with an obvious dynamic recrystallization process, which results in the formation of a dynamic recrystallization structure layer between the rich α_2_ phase layer and the core alloy following the tear. Removing the rich α_2_ phase layer and observing the dynamic recrystallization layer, Fig. 3(c) shows that the B2 phase grain size is small, with a notable softening effect. This reduces the stress on the rich α_2_ phase layer following the tear, and the stress is not sufficient to cause a second tear in the rich α_2_ phase. The B2 phase dynamic recrystallization layer becomes a transitional layer between the rich α_2_ phase layer and the internal core, coordinating the deformation of the two layers. The mechanism for this process is shown in Fig. 4.

## Conclusions

This paper reports the presence of tear strips on the surface of a Ti-22Al-25Nb alloy sheet stretched at 960 °C. The test piece surface shows a “bamboo” shape. The formation mechanism involves a rich α_2_ phase layer formed on the alloy surface at a thickness of 70 μm. This layer has high hardness and poor plasticity and is easy to tear during large deformations. Because the rich α_2_ phase layer restricts the deformation of the neighbouring B2 substrate, the B2 substrate layer experiences large shear stress. A layer of B2 phase dynamic recrystallization forms between the rich α_2_ phase layer and the core alloy after tearing, which dynamically softens and coordinates the deformation of the rich α_2_ phase layer and the core. Overall, these processes reduce the stress on the rich α_2_ phase layer after tearing and prevent a second tearing event.
